# Reproductive health and access to healthcare facilities: risk factors for depression and anxiety in women with an earthquake experience

**DOI:** 10.1186/1471-2458-11-523

**Published:** 2011-06-30

**Authors:** Jasim Anwar, Elias Mpofu, Lynda R Matthews, Ahmed Farah Shadoul, Kaye E Brock

**Affiliations:** 1 Faculty of Health Sciences, the University of Sydney, East Street, Lidcombe, NSW, 1825, Australia; 2 World Health Organization, Main Country Office, UNOCA Compound, Kabul, Afghanistan

**Keywords:** Reproductive Health, Mental Health, Disaster, Depression, Anxiety, Earthquake, Access to Health Facilities, Pakistan

## Abstract

**Background:**

The reproductive and mental health of women contributes significantly to their overall well-being. Three of the eight Millennium Development Goals are directly related to reproductive and sexual health while mental disorders make up three of the ten leading causes of disease burden in low and middle-income countries. Among mental disorders, depression and anxiety are two of the most prevalent. In the context of slower progress in achieving Millennium Development Goals in developing countries and the ever-increasing man-made and natural disasters in these areas, it is important to understand the association between reproductive health and mental health among women with post-disaster experiences.

**Methods:**

This was a cross-sectional study with a sample of 387 women of reproductive age (15-49 years) randomly selected from the October 2005 earthquake affected areas of Pakistan. Data on reproductive health was collected using the Centers for Disease Control reproductive health assessment toolkit. Depression and anxiety were measured using the Hopkins Symptom Checklist-25, while earthquake experiences were captured using the Harvard Trauma Questionnaire. The association of either depression or anxiety with socio-demographic variables, earthquake experiences, reproductive health and access to health facilities was estimated using multivariate logistic regression.

**Results:**

Post-earthquake reproductive health events together with economic deprivation, lower family support and poorer access to health care facilities explained a significant proportion of differences in the experiencing of clinical levels of depression and anxiety. For instance, women losing resources for subsistence, separation from family and experiencing reproductive health events such as having a stillbirth, having had an abortion, having had abnormal vaginal discharge or having had genital ulcers, were at significant risk of depression and anxiety.

**Conclusion:**

The relationship between women's post-earthquake mental health and reproductive health, socio-economic status, and health care access is complex and explained largely by the socio-cultural role of women. It is suggested that interventions that consider gender differences and that are culturally appropriate are likely to reduce the incidence.

## Background

Mental and reproductive health are significant factors that contribute to the overall well-being of women [1-3]. Mental disorders are associated with loss of healthy years of life for women aged 15 - 44 years, a time which spans their reproductive years [4]. They make up three of the ten leading causes of disease burden in low- and middle-income countries [4]. Among mental disorders, depression and anxiety are two of the most prevalent in the general population [5]. Depression is the leading cause of disease burden for women in both high-income and low and middle-income countries [4] with a reported lifetime prevalence of 14% to 21% [6].

According to DSM-IV-TR depression comes under mood disorders while generalized anxiety is classified under anxiety disorders [7]. For both conditions, a stressor is required. Women are more prone to developing depression and anxiety than men. Furthermore, most women experience depression and anxiety during their reproductive years and relationships between depression, anxiety, and the reproductive health of women are identified in the literature [8]. For example, significantly high rates of anxiety have been reported in women attending Sexually Transmitted Diseases (STDs) clinics [9,10]. Similarly, depression has been associated with untoward pregnancy outcomes such as preterm births [11,12]. Co-morbidity of depression with anxiety has also been reported in a number of studies; for example, a WHO multi-center study of co-morbidity found the effects of depression are more debilitating when it occurs alongside anxiety [13].

Several studies have shown that mental health disorders such as post-traumatic stress disorder (PTSD), depression and anxiety are common following natural disasters, including earthquakes [14,15]. Prevalence of depression following an earthquake varied from 9% in Turkey [16] to 79% in Greece [17], while prevalence of anxiety varied from 5% in Italy to 60% in Armenia [18,19]. Bonanno et al. reported that, up to 30% of survivors of a natural disaster had experienced mental health problems. The context in which any particular disaster occurs will influence the psychological morbidity and its outcomes, as well as personal factors such as exposure to the traumatic events, social support and resilience [20]. Mental health following natural disasters are mostly psychosocial in nature and depends upon multiple factors, including poor socio-economic status, lack of family and community support and increased violence [21].

### The Pakistani Context

A systematic review of studies on depression and anxiety in Pakistan showed overall prevalence of depression and anxiety at 34%. The two mental health conditions varied in prevalence among women from 29% to 66% [22]. Similarly, the World Health Organization (WHO) Mental Health Atlas report for Pakistan cited 66% prevalence of depression and 46% anxiety among women [23]. On 8 October 2005, a massive earthquake with a magnitude of 7.6 on the Richter scale struck Pakistan [24]. More than 3.5 million people were affected and displaced in an area spread over 30,000 square kilometres, which had various different health services (District Headquarter Hospitals, Tehsil Headquarters Hospital, Mother and Child Health Services and Basic Health Units). There were more than 73,000 deaths; and homes, markets and businesses were destroyed. Many people were trapped in physically inaccessible areas with no health services (see Additional file 1 for areas affected by the earthquake). Out of 129,000 injured, 17,000 were evacuated using helicopters. Over 70% of health facilities in the area were destroyed. Reproductive health was a major concern because of damaged health facilities and lack of female healthcare providers. Many thousands suffered psychological trauma due to the loss of family members and their livelihood [25]. Organizations on the ground and not destroyed at the time provided basic psychosocial support and referral to the affected primary population (i.e. first care health facilities). Of the displaced, only 275,000 came to settle in the camps while most people remained in tents near their destroyed homes and were, consequently, deprived of psychosocial support [25].

This present study investigated factors that are related to depression and anxiety among women living in the Balakot area affected by the October 2005 earthquake in Pakistan.

### Conceptual framework

This study further builds on the conceptual framework presented by Patel et al. in his study on risk factors for common mental disorders among women of reproductive age in a developing country [5]. In their framework, socioeconomic status, including poverty, was an independent risk factor for common mental disorders. Because of poverty, women in developing countries are more exposed to threatening life events and extreme living conditions. Consequently, they are also more likely to experience chronic diseases. Similarly, association between various reproductive health conditions and diseases with common mental disorders can be better explained in the social context of gynaecological morbidity and gender and power inequalities typical of the role of women in developing countries. Apart from the direct effects of poverty, poor reproductive health and other associated conditions like substance abuse and chronic disease, they also have synergistic effects on each other in the causation of mental disorders.

**Figure 1:**
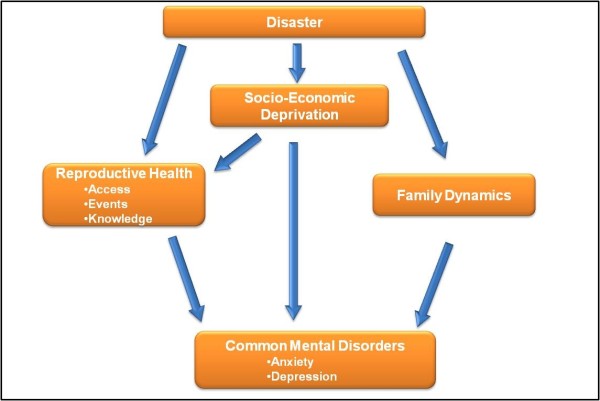
**Conceptual framework: Disasters, Reproductive Health and Common Mental Disorders**.

We adapted the Patel et al.'s conceptual framework [5] to our own (Figure 1) because of the fact that both man-made and natural disasters can cause socio-economic deprivation by destruction of houses and businesses, and loss of employment opportunities. These adverse events affect the reproductive health of women. Socio-economic deprivation is associated with poor reproductive health of women and several chronic diseases, including mental health disorders. The burden from treating mental health problems from disasters adds to the health care burden, and which becomes quite acute in the context of the health of the developing world.

A large number of studies explained the association of adverse reproductive health events with mental disorders by limited access to healthcare facilities [26]. In conservative societies women may experience difficulty in accessing health facilities due a number of factors including lack of female healthcare providers and the cultural norm that women must have an escort for consulting healthcare [26,27]. All these factors make women more vulnerable to the worst effects of any disaster.

### Goals of the study

This study explored the socio-contextual factors that explained depression and anxiety among women of reproductive age (15-49 years) affected by the October 2005 earthquake in Pakistan. It also investigated social-demographic, reproductive health and earthquake experiences related to depression and anxiety. Specific research questions were:

1. What are the family or individual factors related to depression and anxiety among women of reproductive age living in an earthquake-affected area?

2. How are adverse reproductive health events, knowledge of contraceptive methods; and access to health facilities associated with depression and anxiety in a population affected by earthquake?

## Methods

### Participants and Setting

A cross-sectional randomized survey was conducted in tehsil Balakot, one of the two tehsils of District Mansehra, which is in the north of Pakistan in Hazara region. For convenience of sampling, those areas that were provided with the services of the Lady Health Workers (LHWs) programme were included in this study. LHWs are employees of the National Program for Family Planning and Primary Health Care and they are required by the Executive District Health Office to maintain a list of all women in their area. For the purpose of this study LHWs prepared a list of all the women aged 15 - 49 years in stated regions of Hazara affected by the earthquake and covered by LHWs (see Additional file 2). The LHWs identified and removed from their lists women who did not meet the selection criteria during their routine visit to the assigned areas. Exclusion criteria included women who were not married or were widows or were infertile at the time of the earthquake. As the exact proportion of displaced people was not known, 50% exposure was used as an estimate when calculating the sample size. Using an alpha of 0.05 and 97% power, the sample size estimate was 385. With an estimated 10% dropout rate, an initial estimate of sample size of 425 was used. A random sample of 425 was selected from the list of 16,234 eligible women recorded by the 137 LHWs working in tehsil Balakot.

### Instruments

The study used three standardized questionnaires; the CDC Reproductive Health Assessment Toolkit (RHAT) [28], the Harvard Trauma Questionnaire (HTQ) [29], and the Hopkins Symptom Checklist-25 (HSCL-25) [30-32]. The translation process was performed by the principal investigator (first author) and the questionnaire was then back translated for clarity by another Urdu speaking professional in the team. None of the HTQ or HSCL questions were adapted; they were translated literally into Urdu, as after piloting there appeared to be no need for adaptation. We also piloted twenty questionnaires with interviewers in a realistic setting. There were a few problems identified in understanding the pregnancy table, which required better explanation to the interviewers. However, no culturally sensitive issues arose from the questionnaires.

### The CDC Reproductive Health Assessment Toolkit (CDC-RHAT)

We adopted the CDC-RHAT developed by Division of Reproductive Health at the Centers for Disease Control and Prevention, Atlanta. It captured data on the reproductive health status of women affected by conflicts and provided information on basic demographics, pregnancy history, safe motherhood, family planning, sexual history, sexually transmitted infections and gender-based violence. The CDC-RHAT has been successfully applied in Afghanistan, Pakistan and North Uganda [28].

### The Hopkins Symptom Checklist-25 (HSCL-25)

We used the HSCL-25 to assess depression and anxiety in the women affected by the earthquake. It comprised a ten-item subscale for anxiety and a 15-item subscale for depression. It measured anxiety and depression-related symptoms using a four point rating scale (1 = Not at all, 2 = A little, 3 = Quite a bit, 4 = Extremely). The HSCL-25 has been translated into numerous languages including Urdu [33]. The HSCL-25 has been used in a number of international studies in traumatized and post-conflict populations [30-32]. It is very reliable in detecting depression and anxiety [29,34-37]. Internal consistency for the depression subscale was found to be 0.88 and for the anxiety subscale was 0.76 in Tanzania [38]. Another study in China showed internal consistency of the scale to be 0.97 for HSCL-25 total scores [39]. Intra-rater reliability was found to be higher than 0.98 in an Indo-Chinese version used for refugees [30].

### The Harvard Trauma Questionnaire (HTQ)

We used the HTQ to capture information about earthquake events and experiences of living in camps and tents and to asses PTSD [29]. The HTQ has been translated and applied into many languages [37]. It has very high reliability (internal consistency) ranging from 0.86 to 0.94 [29,40-44]. This study is part of larger project on mental and reproductive health following earthquake disaster. Only earthquake experiences and events were included in this study.

### Procedure

Eight trained women interviewers informed potential participants about the survey, its risks and benefits, and that the survey was completely voluntary. Informed written consent was obtained before the interview. The interviews were conducted between 1 September 2009 and 31 October 2009. Ethical clearance was obtained from the Pakistan Medical Research Council (ref. no. F. 4-87/NBC-20/RDC/09/3981), and the University of Sydney (ref. no. 05-2009/11727).

### Data Analysis

Initially descriptive (percentages and means) and analytical (students T test, Pearson's correlation coefficients and chi-square) analyses were performed. Secondly, logistic regression was used to assess risk factors for depression and anxiety [45]. For the purpose of this paper we categorized depression and anxiety as a binary outcome (present/absent) producing risk estimates as odds ratios (OR) and 95% Confidence Intervals (95% CI). This binary outcome was based on the cut-off score recommended in the HSCL-25 manual [34]; participants above the cut-off score of 1.75 were classified as cases i.e. those at risk of depression and anxiety. In order to assess risk determinants, the association of socio-demographic, earthquake experiences and reproductive health factors with depression and anxiety was initially assessed by chi-square and reported if p < 0.05 or OR > 2.0 (Model 1). Risk estimates were assessed in three domains: socio-demographic, earthquake experiences and reproductive health. A second model included mutual adjustment for confounding effects within each domain. A third model consisted of significant predictors mutually adjusted by significant factors of socio-demographic, earthquake experiences and reproductive health, excluding access to health facilities. A fourth model included access to health facilities along with other significant factors.

In the domain of socio-demographic variables, higher age group, having a husband who never attended school, women who had not lived with their husband in past 12 months, being a victim of gender violence by the women's husband or a family member, and having had a husband who lost his job or family business were included as potential predictors. In the domain of earthquake experiences, variables included were: having separated for more than two days from relatives, and having slept for more than one night at a tent village. These variables were entered as predictors of depression and anxiety. In the domain of reproductive health, variables included were: having ever had a stillbirth, having had an abortion in the last four years, having had complications with pregnancy in the last four years, having had abnormal vaginal discharge, genital ulcers, or having heard about STDs in the past 12 months, having ever heard about contraceptives (injectable, vasectomy and rhythm methods), and having difficult access to health facilities were included as potential predictors of depression and anxiety. Having difficult access to health facilities was defined as the nature of the physical location that actually inhibited access to health-care facilities, for example long distances, high mountains, or lack of infra structure (i.e., road or rail availability). Data were analyzed using the SPSS version 17 (Statistical Package for the Social Sciences, SPSS Inc., Chicago, Illinois).

## Results

Out of the 425 initially contacted, 387 women participated in this study (91%). The mean age of the participants was 33.2 years (SD ± 7.2, range 17-49 years) and that of non-responders (9%) was 32.6 years (SD ± 7.2, range 21-48 years). Almost all the women (360) were from rural areas (97%). Eighty-three percent of households were headed by the husband of the participant and only 3% by the participant themselves. The remaining 14% were headed by another member of the participant's family. Socio-demographic characteristic of the study sample are presented in Table 1.

**Table 1:** Sample characteristics of women affected by the October 2005 earthquake in Pakistan (N = 387)

Socio-demographics	N	%	Education	N	%	Family characteristics	N	%
**Age**			**Attended school**			**Head of household**		
35-49 years	157	40.6	No	245	63.3	Respondent herself	15	3.9
15-34 years	230	59.4	Yes	142	36.7	Husband	322	83.2
**Location**			**Level of education**			Other family relatives	50	12.9
Rural	360	93.0	No education	245	63.3	**Current relationship status**		
Urban	27	7.0	Primary school or less	68	17.6	Living with husband	385	99.5
**Current living status**			Middle school and higher	74	19.1	Not living with husband	2	0.5
Temporary shelter/tent	54	14.0	**Husband attended school**			**Lived with husband in last 12 months**		
Own house	333	86.0	Yes	278	71.8	Yes	379	97.9
**Husband job/work**			No	109	28.2	No	8	2.1
Government employee	59	15.2	**Husband education**	109	28.2	**Husband had other wives**		
Business	70	18.1	No education	65	16.8	Yes	13	3.4
Labourer/farmer	246	63.6	Primary school or less	60	15.5	No	373	96.6
Unemployed	12	3.1	Middle school					
			High school and higher	153	39.5			

Ninety-eight percent of the participants became pregnant at least once. Ten percent were currently pregnant. Among those currently pregnant, 23% were pregnant for the first time and 87% had antenatal care mostly provided by doctors. Twenty-six percent of those who had been 'ever pregnant' had experienced the death of a child who was born alive, 7% had experienced a stillbirth and 26% had had an abortion. Twenty-two percent of the 'ever pregnant' women had complications with their pregnancies (before delivery, during labour and post-delivery), 8% had had an abortion and 3% had congenital anomalies in the last four years after the earthquake.

### Risk factors (Predictors) for depression and anxiety

Our study found 63% of the earthquake-affected women had anxiety and 54% had depression. This rate is higher than findings of other studies on the general population. A systematic review by Mirza reported 34% prevalence of depression and anxiety [22]. A recent study conducted in rural population of Pakistan estimated prevalence of depression and anxiety as 43% [46]. Another population-based study of rural areas showed 57% prevalence of depressive disorders [47]. To our knowledge, there is no previous population based study on the earthquake-affected women, with which we can compare our results.

We reported on the effects of socio-demographic factors, earthquake experiences, reproductive health, and access to health facilities as risk factors for depression and anxiety.

### Socio-demographic factors

Among the significant socio-demographic factors, a husband's lower education and having a husband who lost his job or family business were associated with depression. A husband's lower education and having experienced gender violence were associated with anxiety (Table 2, Model 1). Women whose husbands had little formal education were between one and half to two times more at risk of depression and anxiety compared to women whose husbands had a higher level of formal education (OR: 1.5; 95% CI: 1.0-2.3; OR: 1.6; 95% CI: 1.0-2.50; for depression and anxiety respectively). Women whose husbands lost their job or family business were at twice the risk of depression (OR: 1.6, 95% CI: 1.0-2.4). Women who were subjected to gender violence were at two-fold risk of having anxiety compared to those not being exposed to gender violence (OR: 2.2; 95% CI: 1.1-4.3).

**Table 2:** Association of socio-demographic, earthquake experiences and reproductive health factors with depression and anxiety

		HSCL-25 Scores					HSCL-25 Scores		
		Depression		Anxiety		Depression		Anxiety	
		
Variables	Total Sample %(N=387)	1.0-1.74 %(N=179)	≥ 1.75 %(N=208)	1.0-1.74 %(N=144)	≥ 1.75 %(N=243)	Crude OR [95% CI]	Adjusted OR [95% CI] †	Crude OR [95% CI]	Adjusted OR [95% CI] †
**SOCIO-DEMOGRAPHIC FACTORS**									
**Age**									
35-49	59.4	42.5	38.9	45.8	37.4	1.0	-	1.0	-
15-34	40.6	57.5	61.1	54.2	62.6	1.2 [0.8-1.7]		1.4 [0.9-2.2]	
**Husband ever attended school**									
Yes	71.8	67.7	75.5	66.0	75.3	1.0	1.0	1.0	1.0
No	28.2	32.4	24.5	34.0	24.7	1.5 [1.0-2.3]	1.5 [0.9-2.3]	1.6 [1.0-2.5]	1.5 [1.0-2.5]
**Lived with husband in last 12 months**									
Yes	97.9	98.9	97.1	98.6	97.5	1.0	-	1.0	-
No	2.1	1.1	2.9	1.4	2.5	2.6 [0.5-13.1]		1.8 [0.4-9.0]	
**Subjected to gender violence** **by husband or family members**									
No	86.6	88.8	84.6	91.7	83.5	1.0	-	1.0	1.0
Yes	13.4	11.2	15.4	8.3	16.5	1.5 [0.8-2.6]		2.2 [1.1-4.3]	2.2 [1.1-4.3]
**Lost family business or job**									
No	58.4	64.2	53.4	61.1	56.8	1.0	1.0	1.0	-
Yes	41.6	35.8	46.6	38.9	43.2	1.6 [1.0-2.4]	1.6 [1.0-2.4]	1.2 [0.8-1.8]	
**EARTHQUAKE EXPERIENCES**									
**Separated for > two day** ** from family members**									
No	74.9	83.8	67.3	83.3	70.0	1.0	1.0	1.0	1.0
Yes	25.1	16.2	32.7	16.7	30.0	2.5 [1.5-4.1]	2.5 [1.5-4.0]	2.2 [1.3-3.6]	1.9 [1.1-3.3]
**Saw neighbours or friends** **or family members died**									
No	71.8	74.3	69.8	78.1	68.2	1.0	-	1.0	1.0
Yes	28.2	25.7	30.2	21.9	31.8	1.3 [0.8-2.0]		1.7 [1.0-2.7]	1.4 [0.9-2.3]
**Slept > one night at a tent village**									
Yes	24.0	20.7	26.9	18.1	27.6	1.0	1.0	1.0	1.0
No	76.0	79.3	73.1	81.9	72.4	1.4 [0.9-2.3]	1.3 [0.8-2.1]	1.7 [1.0-2.9]	1.7 [1.0-2.8]
**REPRODUCTIVE HEALTH FACTORS**									
**Ever had stillbirth**									
No	93.1	4.0	9.5	95.8	91.5	1.0	1.0	1.0	-
Yes	6.9	96.0	90.5	4.2	8.5	2.5 [1.0-6.2]	2.4 [1.0-6.0]	2.1 [0.8-5.4]	
**Abortions in last four years** **after earthquake **^**a**^									
No	92.3	95.5	89.6	96.5	89.8	1.0	1.0	1.0	1.0
Yes	7.7	4.5	10.4	3.5	10.2	2.5 [1.1-5.7]	2.4 [1.0-5.8]	3.1 [1.2-8.3]	3.7 [1.3-10.6]
**Complications with pregnancy** **in last four years **^**b**^									
Yes	78.1	82.6	74.1	84.3	74.0	1.0	-	1.0	1.0
No	21.9	17.4	25.9	15.7	26.0	1.7 [0.9-3.1]		1.9 [1.0-3.6]	1.5 [0.8-2.9]
**Had abnormal vaginal discharge or** **genital ulcers or heard about STDs**									
No	60.2	68.2	53.4	72.9	52.7	1.0	1.0	1.0	1.0
Yes	39.8	31.8	46.6	27.1	47.3	1.9 [1.2-2.9]	1.6 [1.0-2.4]	2.4 [1.6-3.8]	1.7 [1.0-3.0]
**Ever heard of contraceptives** **(Injectables, vasectomy, rhythms)**									
No	10.9	15.6	6.7	17.4	7.0	1.0	1.0	1.0	1.0
Yes	89.1	84.4	93.3	82.6	93.0	2.6 [1.3-5.1]	2.6 [1.3-5.4]	2.8 [1.5-5.4]	2.9 [1.2-7.0]
**Access to health facilities**									
Easy access	74.4	90.5	60.6	92.4	63.8	1.0		1.0	
Difficult access	25.6	9.5	39.4	7.6	36.2	6.2 [3.5-11.0]	-	6.9 [3.5-13.4]	-

Abbreviations: HSCL-25, Hopkins Symptom Checklist-25; OR, Odds Ratios; CI, Confidence Interval; STDs, Sexually Transmitted Diseases.

†: Odds ratios mutually adjusted within each domain of socio-demographic, earthquake experiences and reproductive health.

All variables with a P value of 0.05 or less in the univariate analysis were entered into the multivariate logistic regression analysis.

a Women who ever got pregnant; N = 378; Depression (N): Below cut-off = 177, Above cut-off = 201; Anxiety (N): Below cut-off = 142, Above cut-off = 236.

b Women who delivered in last four year after the earthquake, N = 256; Depression (N): Below cut-off = 121, Above cut-off = 135; Anxiety (N): Below cut-off = 102, Above cut-off = 154.

When mutually adjusted within the domain of socio-demographic factors (Table 2, Model 2), no significant change was observed in the predictive factors for anxiety. When adjusted for depression, a husband having lost his job or business remained significant (OR: 1.6; 95% CI: 1.0-2.4) however, the association between a husband's low level of education and depression became non-significant (OR: 1.5; 95% CI: 0.9-2.3).

Multivariate logistic regression analyses were performed to further investigate associations and to control for possible confounding factors from the other two domains of earthquake experiences and reproductive health factors (Table 3, Model 3). No change was observed in the odds of depression with the risk of a husband losing his job. However, the effect of husband's lower education on the anxiety became non-significant due to the confounding effect of the variable 'having heard about injectable contraceptives'. The reduction in effect was because women who had a husband with a lower level of schooling were three times more likely to be ignorant about injectable contraceptives compared to those whose husbands had a higher level of schooling (OR: 2.7; 95% CI: 1.4-5.0). Similarly, the significant effect of having experienced gender violence was reduced because of the confounding effect of the variable 'having an abnormal vaginal discharge or had genital ulcers' (OR: 1.7; 95% CI: 0.8-3.8). This reduction in effect was because women who had abnormal vaginal discharge or genital ulcers were more than two times at risk of being subjected to gender violence compare to those who did not have abnormal vaginal discharge or genital ulcers (OR: 2.3; 95% CI: 1.3-4.3).

**Table 3:** Multivariate analysis of significant risk factors for depression and anxiety among women of reproductive age with earthquake experience (N = 387)

	Depression		Anxiety	
	
Factors	Adjusted **OR [95% CI] **†	Adjusted **OR [95% CI] **†‡	Adjusted **OR [95% CI] **†	Adjusted **OR [95% CI] **†‡
**Husband ever attended school**				
Yes	-	-	1.0	1.0
No			1.4 [0.9-2.3]	1.4 [0.8-2.3]
**Subjected to gender violence by husband or family members**				
No	-	-	1.0	1.0
Yes			1.7 [0.8-3.6]	1.3 [0.6-2.9]
**Lost family business or job**				
No	1.0	1.0		
Yes	1.6 [1.0-2.4]	1.6 [1.0-2.5]	-	-
**Separated for > two days from family members**				
No	1.0	1.0	1.0	1.0
Yes	2.5 [1.5-4.2]	1.5 [0.8-2.6]	2.3 [1.3-3.9]	1.3 [0.7-2.4]
**Slept more than one night at a tent village**				
Yes			1.0	1.0
No	-	-	1.5 [0.9-2.7]	1.3 [0.7-2.2]
**Ever had stillbirth**				
Yes	1.0	1.0		
No	2.5 [1.0-6.3]	2.4 [0.9-6.2]	-	-
**Abortions in last 4 years after earthquake**				
No	1.0	1.0	1.0	1.0
Yes	2.3 [1.0-5.7]	2.4 [1.0-5.9]	2.7 [1.0-7.7]	2.9 [1.0-8.4]
**Had vaginal discharge or genital ulcers or heard about STDs**				
No	1.0	1.0	1.0	1.0
Yes	1.7 [1.1-2.6]	1.5 [0.9-2.4]	1.9 [1.2-3.0]	1.8 [1.1-2.9]
**Ever heard of contraceptives (injectable, vasectomy, rhythms)**				
No	1.0	1.0	1.0	1.0
Yes	2.7 [1.3-5.8]	2.7 [1.2-5.9]	2.6 [1.3-5.5]	2.5 [1.2-5.4]
**Access to health facilities**				
Easy access		1.0		1.0
Difficult access	-	5.0 [2.6-9.4]	-	5.6 [2.6-11.9]

Abbreviations: OR, Odds Ratio (adjusted); CI, Confidence Interval; STDs, Sexually Transmitted Diseases.

Only statistically significant variables (p < 0.05) following multivariate analysis within each domain of socio-demographic, earthquake experience and reproductive health were included in this analysis.

**† **Adjusted odds ratios without access to health facilities.

†‡ Adjusted odds ratios with access to health facilities.

### Earthquake experiences

Being separated from a family member was a strong risk factor for both depression and anxiety. Women who were separated for two days or more from any family members were two to three times at risk of depression and anxiety (OR: 2.5; 95% CI: 1.5-4.1; OR: 2.2; 95% CI: 1.3-3.6, respectively) compared to those who were not separated from any family members. Additionally, women who witnessed the death of a friend, neighbour or relative and slept more than one night at a tent village were significantly at risk of developing anxiety (p < 0.05). Women who witnessed the death of a friend, neighbour or relative were two times more likely to have anxiety compared to those who had not witnessed death (OR: 1.7; CI: 1.0-2.7), while women who had slept at a tent village were two times more likely to experience anxiety compared to those who had not slept at a tent village (OR: 1.7; 95% CI: 1.0-2.9).

When mutually adjusted within the domain (Table 2, Model 2), no significant change was observed in the risk of depression and anxiety due to having separated from a family member or risk of anxiety due to having lived in a tent. However, the risk of anxiety due to having witnessed the death of a friend, neighbour or relative became non-significant (OR: 1.4; 95% CI: 0.9-2.3). This result was due to the confounding effect of being separated from a family member. Women who were separated from any family member were three times more likely to witness the death of a friend, neighbour or a family member compared to those who had not witnessed deaths of a friend, neighbour or a family member (OR: 3.4; 95% CI: 2.1-5.5). We hypothesize that these women may be more likely to have been living in areas of severe destruction where the possibility of separation from family and witnessing death would be high.

When those factors that remained significant were entered in a final multivariate regression model (Table 3, Model 3), having been separated from a family member remained a significant predictive factor for depression and anxiety. The risk of anxiety among women who had slept for two or more days in a tent village became non-significant due to the confounding effect of separation from family members. This was because women who were separated from any family member were two times more likely to have slept in a tent village compared to those who were not separated from any family member (OR: 1.9; 95% CI: 1.1-3.1).

### Reproductive health factors

Having had an abortion, having had abnormal vaginal discharge, genital ulcers or whether participants had ever heard about STDs, and having heard about contraceptives were significant risk factors for depression and anxiety (Table 2). In addition, women who had experienced a stillbirth were at risk of depression and women who had complications with pregnancy were at risk of anxiety. Women who had abortions in the last four years were two to three times more at risk of depression and anxiety compared to those who did not have an abortion in last the four years (OR: 2.5, 95% CI: 1.1-5.7; OR: 3.1, 95% CI: 1.2-8.3, for depression and anxiety respectively). Women who had abnormal vaginal discharge or genital ulcers or had heard about STDs in the last year were at twice the risk of depression and anxiety compared to those not having abnormal vaginal discharged or genital ulcers or not having heard about STDs (OR: 1.9, 95% CI: 1.2-2.9; OR: 2.4, 95% CI: 1.6-3.8; for depression and anxiety respectively). Further, women who had heard of contraceptive methods (injectable-vasectomy-rhythm method) were at two and half-fold risk of depression and anxiety (OR: 2.6, 95% CI: 1.3-5.1; OR: 2.8, 95% CI: 1.5-5.4, respectively). Women who had experienced a stillbirth were at two times the risk of depression (OR: 2.5; 95% CI: 1.0-6.2), while women who had complications with the pregnancy were at two times the risk of having anxiety compared to those who had no complications with the pregnancy in the last four years after the earthquake (OR: 1.9; 95% CI: 1.0-3.6).

After adjusting for the possible confounding effects of other variables within the domain of reproductive health (Table 2, Model 2), no significant change was observed in the odds of depression due to having had a stillbirth, having had an abortion, having had an abnormal vaginal discharge or genital ulcers, or having knowledge about contraceptive methods. However, among the risk factors for anxiety it was noted that complications with pregnancy became non-significant, with experience of abortion (OR: 1.5; 95% CI: 0.8-2.9). This happened due to the confounding effect of abortions. This is because women who had abortions in the last four years were three times at risk of developing complications with pregnancy compared to those who had no abortion (OR: 2.5; 95% CI: 1.1-5.6). No significant change was observed in the risk of anxiety due to having had an abortion, abnormal vaginal discharge or genital ulcers and having heard of contraceptives (Table 2).

Those variables that remained significant after mutual adjustment within the domain of reproductive health were entered in the final model with variables from the other two domains (Table 3, Model 3). No significant change was observed on the predictors for depression due to having had an abortion, stillbirth, abnormal vaginal discharge or genital ulcers, and having heard about contraceptive methods. However, the effects of abortion on anxiety decreased from four to three times (OR: 2.7; 95% CI: 1.0-7.7). This decrease was due to the confounding effect on abortion of having experienced gender violence; women who were subjected to gender violence were four times at risk of anxiety compared to those not subjected to gender violence (OR: 3.9; 95% CI: 1.7-8.8). No change was observed in the odds of abnormal vaginal discharge or genital ulcers and use of injectable contraceptives on anxiety.

### Access to health facilities

Limited access to health facilities was found to be a strong predictive factor for depression and anxiety. Women who had difficult access to health facilities were six to seven times at greater risk of depression and anxiety compared to those who had easy access to health facilities (OR: 6.2, 95% CI: 3.5-11.0; OR: 6.9, 95% CI: 3.5-13.4; for depression and anxiety respectively). When access to a health facility was added to the final model (Table 3, Model 3) as a confounder along with other factors from the three domains (socio-demographic, earthquake experiences and reproductive health) the significant predictive effects on depression of having separated from a family member, having had a stillbirth and having had an abnormal vaginal discharge or genital ulcers disappeared (Table 3, Model 3).

Thus, lack of access to healthcare facilities was the major determinant of depression and not reproductive health events, that is, reproductive health events were exacerbated by a lack of access to healthcare facilities. Women who were separated from any family member, had a stillbirth, or had abnormal vaginal discharge or genital ulcers were two to seven times more likely to have difficult access to health facilities compared with women who did not experience these situations (OR: 7.3; 95% CI: 4.4-12.2; OR: 1.7; 95% CI: 0.7-4.0; OR: 1.9; 95% CI: 1.2-3.0 for separation, stillbirth and abnormal vaginal discharge or genital ulcers respectively). Factors that remained significant were: abortions, lost job or family business and knowledge and use of contraceptive methods. Effects of separation from family on anxiety also disappeared. This was because women who were separated from any family member were seven times more likely to have difficult access to health facilities compared with women who had easy access to health facilities (OR: 7.3; 95% CI: 4.4-12.2).

## Discussion

This study investigated the association of family and individual factors, reproductive health events, and access to health facilities with depression and anxiety among women of reproductive age following earthquake experience. The discussion is organized around the research questions.

In regard to family or individual factors that are related to depression and anxiety among women of reproductive age living in an area affected by earthquake, our study found that socio-economic status had a strong association with depression. Women whose husbands were not educated and had lost their job or family business were more likely to be depressed when compared with those whose husbands were more educated and had not lost a job or family business. This is consistent with the findings of Mirza et al. from a systematic review of the literature on depression and anxiety in Pakistan where women's low socio-economic status and poor relationships with their husband and in-laws were found to be important factors for depression and anxiety [22]. Other studies by Patel et al. have also confirmed the association of low socio-economic status with common mental disorders including depression and anxiety [5,48].

We found that women separated from family members following an earthquake had an increased risk of depression and anxiety. Family dynamics play a significant role in the mental health of women in the cultural context of Pakistan [49]. Women are dependent on their husbands or other family members for shelter, security and food. Therefore, the sense of insecurity due to separation may adversely affect the mental health of women. The extreme stress of not knowing the whereabouts of her loved ones may also have negatively impacted the mental health of women [50].

An interesting finding was that the strong association of having experienced gender violence with anxiety became non-significant after adjustment for confounding factors, in this case ever having heard of STDs. We hypothesise that knowledge of STDs is a proxy for socio-economic level. Similar findings were also reported by Patel et al. in their study conducted in India, which showed a strong association of gender violence with common mental disorders that also became non-significant after adjustment for socio-economic status [5]. He found poverty and marital status (widows) confounded the association of gender violence with risk of common mental disorders. Further in-depth studies are needed to further explore these intertwined associations.

We found a strong association between reproductive health events (including stillbirths, abortions, and abnormal vaginal discharge or genital ulcers) and depression and anxiety. Several studies suggest that psychological stress and lack of access to health care following a disaster may adversely affect pregnant women and infants [51]. Poor pregnancy outcomes including increased rate of spontaneous abortion [52], low birth weight babies [53], preterm delivery [54], change in sex ratios [55], retardation of fetal brain development [56] and reduction of breast milk [57], have previously been found to be associated with increased psychological stress following natural disasters. Our findings of an association of abnormal vaginal discharge and genital ulcers with depression and anxiety are consistent with those of Shujuan et al. which followed the 2008 earthquake in China [58]. They suggested that immune suppression stress induced by socio-economic deprivation following earthquake as a possible cause of increased frequency of genital tract infections, menstrual disorders and sexual dysfunction [58]. Osborn et al. found that 50% of patients attending STD clinics had significant anxiety, and that the anxiety may be due to somatization in response to psychosocial stress [59]. Other studies also found associations between abnormal vaginal discharge and increased risks of depression and anxiety [5,10]. We hypothesise that distressing sexual relationships due to a women's genital ulcers and vaginal discharge may increase her levels of anxiety and depression; this explanation has been reported elsewhere [9,10,59-64].

The higher risk of depression and anxiety associated with knowledge about contraceptive methods is a new finding. One explanation for this is that women who had heard about contraceptive methods were more likely to have used these methods, including injectable contraceptives. Injectable contraceptives contain Depot Medroxy Progesterone Acetate (DMPA). DMPA use may be associated with increased risk of depression and anxiety, which was also supported by Civic at al. in their population based study, who suggested pharmacological effect of this drug (DMPA) as a cause of depression [65]. A similar finding of a positive relationship between oral contraceptives and depression was also observed by Kulkarni et al. in women's use of oral contraceptives [66]. They found higher rates of depression among women using combined oral contraceptives (progesterone + estrogens) compare to non-combined oral contraceptives.

Having limited access to health facilities was found to be the strongest predictor for depression and anxiety, a finding that supports results in other studies that investigate various factors related to women's access to health facilities in Pakistan [27,67]. In conservative societies like Pakistan, women need to seek permission from their husband or other male members to consult a doctor or health facility. Additionally, the lack of female healthcare providers may mean that many women are uncomfortable in discussing matters related to pregnancy, contraception and STDs with male healthcare providers.

### Strengths and limitations

One of the strengths of this study is that it is a comprehensive population based study linking reproductive health status and knowledge with depression and anxiety in women of reproductive age in an earthquake-affected area. In addition, having a relatively large sample size of 387 and with randomization in selecting participants adds to our confidence in our findings.

Being a cross-sectional study design, this study could not establish direct causal relationships between reproductive health outcomes and depression and anxiety. Associations found in this study may indicate that poor reproductive health and poor access to health services leads to poor mental health. Alternatively, poor mental health could be a factor in increasing vulnerability to poor reproductive health (e.g. through altered sexual behaviours) and poor health seeking behaviour. In this instance, the direction of the causal pathway between mental health and reproductive health is not known. Prospective studies are needed to determine causal pathways and in the situation of natural disasters, such as an earthquake, these studies are often not feasible. In the literature, however, others have found an association between poor mental health an maternal morbidity, suicidal tendencies, poor fetal growth, risky behaviours like substance abuse, lower rates of antenatal visits and increased risk of adverse obstetric outcome [68].

It should be noted that participants were recruited from areas that were provided with the service of LHWs from the national programme for family planning and primary healthcare and there may be a sample bias in regards to reproductive health practices. Although the LHWs programme covered 57% of the population living in tehsil Balakot, results cannot be generalized to the general population. Future studies would benefit from the inclusion of a control population that has not been provided with the services of LHWs with which to compare results. In addition, future studies should also explore the impact of other possible factors on women's mental health, including the effects of time since the earthquake and religious extremism in such societies.

We used a cut-off score of 1.75 on the HSCL-25. This cut-off point has yielded very high sensitivity and specificity when applied to an Indonesian population of refugees [30]. Although the HSCL-25 has been widely used as a screening instrument to detect depression and anxiety in many cultures and languages including Urdu [29,33,35,37,38,50,69], it was not practicable to test the specificity and sensitivity of the cut-off point of 1.75 against a clinically-based DSM-IV or ICD-10 diagnosis in this population, which is the gold standard. However, it must be emphasized that this is the first large scale study conducted on women of reproductive age (15-49 years) following the October 2005 earthquake in Pakistan.

This study did not measure pain, physical dysfunction or disability and it is possible that these variables influenced the reports of depression and anxiety. Further research is needed to replicate and extend these findings and to address the limitations of the current study. It must also be stressed that some of the more novel associations found in our analyses could be due to multiple testing and/or by chance and as such should be viewed with caution until found in future data sets with larger sample sizes. Instruments used to assess mental disorders following earthquakes or other large-scale traumatic events in Pakistan should be validated in the relevant clinical and field settings. Future work should use longitudinal study designs to examine the potential predictors of depression and anxiety in greater detail, some of which were identified in our study.

## Conclusion

Women's post-disaster mental health is associated with low socio-economic status, separation from family, poor reproductive health outcomes, knowledge of contraception, and limited access to appropriate health facilities. While the relationships between the variables are complex and in need of longitudinal follow-up study, culturally sensitive studies to clarify, post-disaster effects could reduce the incidence of long-term anxiety and depression in women who survive earthquakes.

## Recommendations

The strong association between health, poverty and access to healthcare should be taken into account in developing training programmes for healthcare providers to provide post-disaster interventions as has been produced recently in a publication concerning a framework for mental health and psychosocial support after the Tsunami by WHO [70]. As was concluded in a recent systematic review not only is more research needed into this area but, in particular, emphasis should be placed on empowering women and reducing gender disparities [71]. The design of post-disaster interventions should be tailored to meet the socio-economic and cultural status of women. Development of regular screening programmes for risk factors identified in this and other well-designed studies, with particular emphasis on physical and social barriers in accessing health facilities may significantly reduce the disease burden due to poor women's mental health after similar disasters in developing countries.

## Competing interests

The authors declare that they have no competing interests.

## Authors' contributions

JA was principal investigator of the study and conceptualized the research. He collected the data and performed analysis, and interpretation of results and drafted the article. KB and EM were primary supervisors of JA, assisted in performing analysis, and provided overall guidance to JA. JA, LM and AS participated in the study design, methodology and ethical clearance. All authors contributed significantly to towards drafting and editing the manuscript. All authors have read and approved the manuscript.

## Pre-publication history

The pre-publication history for this paper can be accessed here:

http://www.biomedcentral.com/1471-2458/11/523/prepub

## Supplementary Material

Additional file 1 **Overview of the Affected Population by the October 2005 Earthquake in Pakistan**. Map of all villages affected by the October 2005 earthquake in Pakistan showing the percentage of affected population and number of affected villages in each teshil of the affected districts. Click here for file

Additional file 2 **Map of all Health Facilities and Internally Displaced Population Camps**. Map showing locations of health facilities and relief camps in tehsil Balakot of district Mansehra following the October 2005 Earthquake Click here for file
