# Supplementary figures and images for: Reproductive health and access to healthcare facilities: risk factors for depression and anxiety in women with an earthquake experience
Source: BMC Public Health. 2011 Jun 30;11:523. doi: 10.1186/1471-2458-11-523 (PMC3146866; doi:10.1186/1471-2458-11-523)

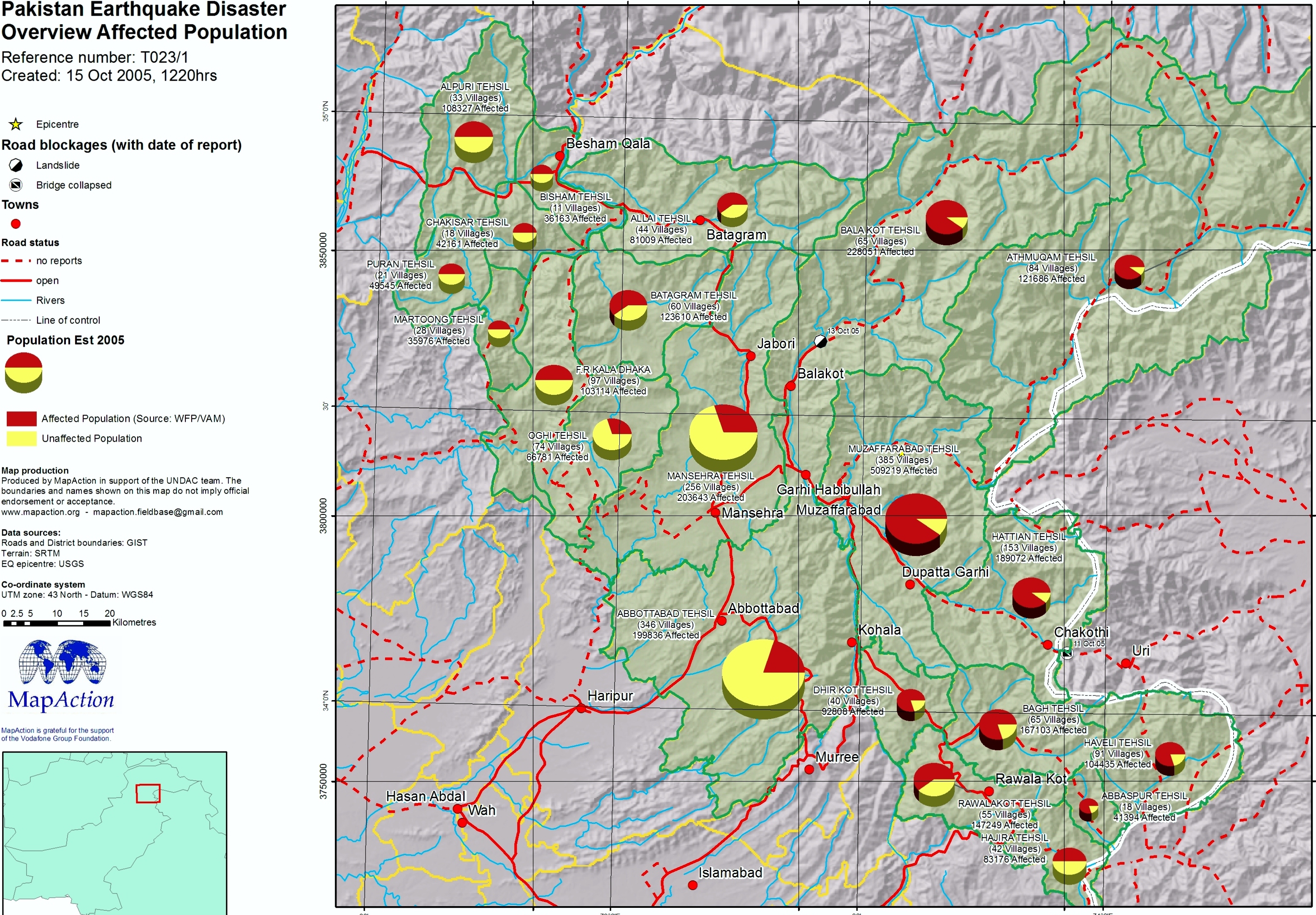

Supplement: Additional file 1 — Overview of the Affected Population by the October 2005 Earthquake in Pakistan. Map of all villages affected by the October 2005 earthquake in Pakistan showing the percentage of affected population and number of affected villages in each teshil of the affected districts. [file 1471-2458-11-523-S1.JPEG]

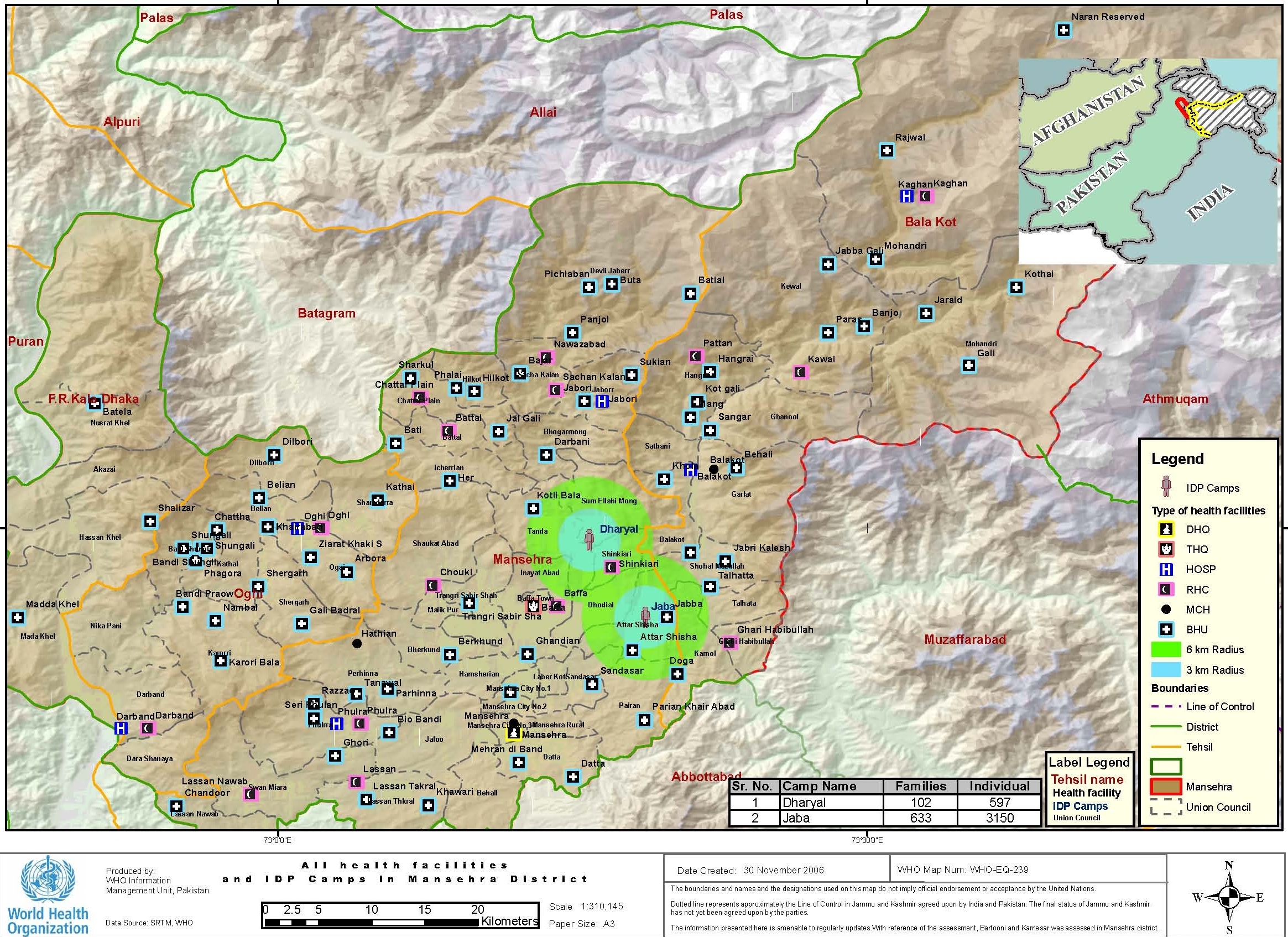

Supplement: Additional file 2 — Map of all Health Facilities and Internally Displaced Population Camps. Map showing locations of health facilities and relief camps in tehsil Balakot of district Mansehra following the October 2005 Earthquake [file 1471-2458-11-523-S2.JPEG]
